# Treadmill Exercise during Pregnancy Decreased Vulnerability to Neonatal Hypoxia-Ischemia through Reducing Inflammation and Increasing Antiapoptotic Gene Expressions and Antioxidant Capacity in Rats

**DOI:** 10.1155/2021/5512745

**Published:** 2021-04-14

**Authors:** Elahe Gorgij, Hamed Fanaei, Parichehr Yaghmaei, Mohammad Reza Shahraki, Hadi Mirahmadi

**Affiliations:** ^1^Department of Biology, Science and Research Branch, Islamic Azad University, Tehran, Iran; ^2^Pregnancy Health Research Center, Zahedan University of Medical Sciences, Zahedan, Iran; ^3^Department of Physiology, Zahedan University of Medical Sciences, Zahedan, Iran; ^4^Infectious Diseases and Tropical Medicine Research Center, Zahedan University of Medical Sciences, Zahedan, Iran

## Abstract

**Background:**

The purpose of present study was to assess the impact of maternal treadmill exercise during pregnancy on inflammation, oxidative stress, expression of Bax and Bcl-2 genes, and brain-derived neurotrophic factor (BDNF) level in neonatal rat brain after the hypoxia-ischemia injury. *Material and Methods*. A total of 24 female Wistar rats were utilized in this research. Two groups are randomly considered for rats: (1) not exercised through pregnancy and (2) exercised during pregnancy. Offsprings were divided into four groups including after delivery: (1) sham, (2) sham/exercise (sham/EX), (3) HI, and (4) HI+exercise. HI was induced in pups at postnatal day 8. Neurobehavioral tests were done seven days after HI induction. Then, the brain tissue was taken from the skull to estimate Bcl-2 and Bax gene expressions, BDNF, cerebral edema, infarct volume, inflammatory factors, oxidative stress, and neurological function.

**Results:**

The BDNF level in the HI+exercise group was considerably higher than the HI, sham, and sham/EX groups. Tumor necrosis factor (TNF-*α*), C-reactive protein (CRP), and the whole oxidant capacity (TOC) levels in the HI group were significantly higher than the sham and sham/EX groups. TNF-*α*, CRP, and TOC levels in the HI+exercise group were significantly lower than the HI group. Total antioxidant capacity (TAC) level in the HI+exercise group was significantly higher than the HI group. Infarct volume and edema percent in the HI+exercise group were significantly lower than the HI group. Neurological function in the HI+exercise group was significantly better than the HI group. Bax expression in the HI+exercise group was significantly lower than the HI group. Bcl-2 expression in the HI+exercise group was significantly higher than the HI group. In the sham group, BDNF, TNF-*α*, CRP, TAC, TOC, edema levels, and neurological function had no significant difference with the sham/EX group.

**Conclusion:**

It appears that the maternal treadmill exercise during pregnancy exerts a supportive impact against neonatal HI brain injury through increasing antioxidant capacity, Bcl-2 expression, and BDNF levels and decreasing inflammation that is resulted in the lower infarct volume and sensorimotor dysfunction.

## 1. Introduction

In cardiovascular, metabolic, endocrine, and musculoskeletal systems of the mother and external elements like nutritional intake, emotional support, and environmental situations, pregnancy induces complex biological modifications that have a significant role to modulate intrauterine milieu and ongoing fetal development [1]. The mother's condition during pregnancy has profound effects on how the fetus grows and develops. Changes in the health of the pregnant mother can affect the condition of important organs of the fetus, such as the cardiovascular organs and the nervous system [1]. Studies have shown that exercise during pregnancy has beneficial effects on the condition of the fetus in various ways, such as increasing the secretion of growth factors [1].

Preventive treatments through pregnancy can be efficient methods modulating the mother-fetus unit. Physical activity through pregnancy can decrease complications in the mother and fetus during the advancement of intrauterine environment that might have permanent impacts on future health of offspring [2]. Exercise for a pregnant mother can reduce maternal complications. It also provides a better intrauterine environment for fetal growth, which will have lasting effects on the future health of the children [2]. Hypoxia-ischemia (HI) is a common cause of brain damage and long-term disability in human infants. Injuries such as sensory, motor, and cognitive impairment may remain with the child for the rest of her/his life [2, 3].

Due to a decrease in brain blood and/or oxygen flow, hypoxic-ischemia (HI) takes place which compromises the oxidative metabolism, resulting in a reducing in energy levels and increased glutamate release, resulting in excitotoxicity damage cascade, metabolic failure, alterations in neuron-glia coupling, and cell death [4, 5]. Neonatal HI occurs during pregnancy, delivery, or postnatal period, which includes decreased oxygenation or/and blood flow to the fetus [4].

Studies have shown that exercise during pregnancy has beneficial effects on the fetal nervous system. These effects include increased secretion of growth factors and increased neurogenesis [4]. It has been shown that exercise training enhances the antioxidant defense system in the brain regions of a rat [6]. Scientific evidences show the favorable effect of physical exercise on the balance between pro- and anti-inflammatory activities in the brain in the face of various disorders [7, 8]. It also enhances the potential of exercise therapy in reducing the risk of neuroinflammation disorders [7]. Inflammatory responses and oxidative stress are important factors in the spread of injury after neonatal hypoxia-ischemia [9].

Considering the fact that exercise can reduce oxidative stress and inflammation, this study evaluated the effect of treadmill exercise during pregnancy on the neonatal brain damage caused by HI in rats.

## 2. Materials and Methods

### 2.1. Animals

Twenty female Wistar rats (200-220 g) were provided from Laboratory Animal Research Center of Zahedan University of Medical Sciences, Zahedan, Iran. The animals were adapted to laboratory environment one week before beginning the experiment. They were kept in standard temperature (21 ± 2°C), on a 12/12 h light/dark cycle, with food and water available ad libitum.

The research protocol was confirmed by Faculty of Medicine Ethics Committee for animal Research of Zahedan University of Medical Sciences, (ethical code: IR.ZAUMS.REC.1399.514).

### 2.2. Experimental Design

Two female Wistar rats were maintained along with one male Wistar rat in a cage to pregnancy induction [9]. The animals' vaginas were tested to find any sperm every morning, and if the test was positive, they were separated and maintained in other cages [9].

Pregnant rats were divided into two groups: (1) the group which not exercised during pregnancy (the pups of the sham and HI groups were chosen from the offsprings of these animals) and (2) the group which daily exercised through pregnancy (pups of the sham/EX and HI+exercise groups were chosen from offsprings of these animals). Offsprings were divided into four groups (20 rats in every group) after delivery: the (1) sham group: surgery was done in this group, and the right common carotid artery (CCA) was not occluded and hypoxia was not induced; (2) sham/EX group: animals of this group were also selected from dams who exercised during pregnancy and the right CCA of the rats was exposed, but not occluded and they did not experience hypoxia; (3) HI group: pups of this group were subjected to right CCA occlusion and exposed to 8% oxygen for 90 minutes; and (4) HI+exercise group: pups of this group were chosen from dams who exercised through pregnancy, and the right CCA of pups was occluded and then exposed to 8% oxygen for 90 minutes.

### 2.3. Exercise Protocol

Rats were trained to move on the treadmill (four-line animal treadmill) through pregnancy. Animals moved on treadmill as follows: (1) at 15 minutes in 5-7 meter/min without a slope; (2) treadmill training in 18 meter/min during 35 min in a slope of zero; (3) after one week, the rats in 18 meter/min during 40 min in a slope of 5°; and (4) the rats were located in the speed of 18 meter/min at 45 min in slope of 10° in the next week [10].

### 2.4. Neonatal Hypoxia-Ischemia Induction

For neonatal HI induction in rat pups, the Rice-Vannucci approach was utilized [9, 11]. To HI induction, 8-day-old pups were anesthetized by ketamine (100 mg/kg) and xylazin (10 mg/kg). Then, the right carotid artery (CCA) was separated from the vagus nerve and surrounding tissue and then CCA permanently occluded with 6.0 silk thread. Also, animals were permitted to recover for one hour (h) and exposed to 8% oxygen for 90 minutes [9]. Neurobehavioral tests were performed seven days after HI induction (postnatal 15^th^ day), and the animals were sacrificed and brain tissues were gathered to measure the expression of Bcl-2 and Bax genes, BDNF, cerebral edema, infarct volume, inflammatory factors, and oxidative stress.

### 2.5. Assessment of Cerebral Edema and Infarction Volume

Pups were anesthetized (ketamine (100 mg/kg) and xylazin (10 mg/kg)) seven days after HI (15-day-old pups), and their heads isolated from the body. The brain was taken from the skull and then put on 10% formalin. Then, 6 micron slices were taken utilizing a rotary microtome (Leica RM 2135, Leica Instruments, Nussloch, Germany). Then, slices were stained with hematoxylin-eosin.

Their images were captured by a scanner (Scanjet, Hewlett-Packard, USA) and assessed utilizing ImageJ software (NIH), and the area of ischemic region was measured. At last, the following formula was utilized to compute ischemic area volume:

Volume of ischemic area [9, 12]:

(1) $$\begin{array}{c} \text{Volume of the ischemic region}=\frac{\left(\text{volume of the contralateral hemisphere}-\text{the volume of the nonischemic region of the ipsilateral hemisphere}\right)}{\text{volume of contralateral hemisphere}}\times 100. \end{array}$$

The following formula was utilized to compute cerebral edema [9, 13]:

(2) $$\begin{array}{c} \text{Edema}=\frac{\;\left(\text{volume of ipsilateral hemisphere}-\text{volume of contralateral hemisphere}\right)}{\text{volume of contralateral hemisphere}}. \end{array}$$

### 2.6. Biochemical Measurements

Furthermore, brain tissue samples were provided and located in a cold buffer (0.1 M phosphate-buffered saline, pH 7.4, involving protease inhibitor cocktail (Roche)); for 15 minutes, it was homogenized and centrifuged at 3500 rpm, and supernatants were gathered. TNF-*α* (Zellbio, Germany), C-reactive protein (CRP) (Zellbio, Germany), BDNF (Zellbio, Germany), total antioxidant capacity (TAC) (Zellbio, Germany), and total oxidant capacity (TOC) (Zellbio, Germany) were measured utilizing special kits.

### 2.7. Bax and Bcl-2 Expression Measurement

Utilizing RNase mini kit (Qiagen, USA), RNA extraction was done based on the manufacturer's instructions and RNA was synthesized from cDNA samples. Also, microtubes were stored at -20°C.

### 2.8. Polymerase Chain Reaction

Polymerase chain reaction (PCR) was utilized for Bcl-2 expression, Bax genes as target genes, and *β*-actin as a structural gene for internal control and confirmation of PCR. PCR was handled by cDNA synthesized due to protocol and utilizing primers (Table 1). Primary denaturation was performed at 95°C for 5 min. Binding temperature for primer Bcl-2, Bax, and *β*-actin was identified at gradient temperature of 56 to 60°C for 30 seconds. The replication temperature for every cycle was 72°C for 20 seconds and for the primers 35 cycles and the latest replication of amplified DNA was performed for 5 minutes at 72°C. PCR product was electrophoresed on 1.5% agarose gel. Gene samples were incubated with the gene marker at 110 V for 30 min, and also, agarose gel was embedded in the dock gel and bands were visualized under UV light docking device (Bio-Rad, USA) and the ratio was identified. Due to the amount of *β*-actin gene of the same group and plotted, the target gene band density of every group was assessed.

### 2.9. Neurobehavioral Tests

#### 2.9.1. Cliff Avoidance Test

On the 15-day-old pups, a cliff avoidance test was carried out in order to evaluate integrity of their motor output and sensory input [9]. The pups were positioned on a platform edge (30 × 30 × 30 cm) with four limbs in a way that their chest touched the platform edge [9]. Besides, the period of withdrawing or turning away from the platform edge was documented. If the pups collapsed from the edge or did not react within 60 seconds, the time would be recorded as the intended period [9].

#### 2.9.2. Negative Geotaxis Test

To put it briefly, this test is a stimulus-bound orientation automatic movement, which tests the neonatal rats' sensorimotor function [9]. A negative geotaxis test was performed for identifying deficits in the proprioceptive and vestibular functions. On a rough surface with a 30° slope, the animal pups were positioned. In addition, each pup's head was located downwards. In addition, the latency to return to an upward direction (180°) was documented. The documented maximum time was 90 seconds [9].

### 2.10. Statistical Analysis

To examine the data, the software GraphPad Prism ver. 8 was utilized. Data analysis was done applying one-way ANOVA and Bonferroni's test. Data was noted as mean ± standard error of mean. *P* < 0.05 was mentioned as significant.

## 3. Results

### 3.1. Biochemical Measurements

#### 3.1.1. The Impacts of Maternal Exercise through Pregnancy on BDNF Levels of Pups after HI

**Figure 1:**
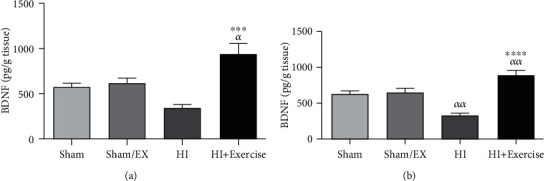
Effect of exercise during pregnancy on BDNF levels after HI in female (a) and male (b) pups brain. HI: hypoxia-ischemia. *^α^P* < 0.05, *^αα^P* < 0.01 as compared with the sham and sham/EX. ^∗∗∗^*P* < 0.001, ^∗∗∗∗^*P* < 0.0001 as compared with the HI. Data are expressed as the mean ± S.E.M. (*n* = 10).

According to Figure 1(a), BDNF levels of female pups were higher in the HI+exercise group compared to the sham and sham/EX groups (*P* < 0.05). Furthermore, in female pups, BDNF levels were higher in the HI+exercise group in comparison to the HI group (*P* < 0.001). Considering Figure 1(b) in male pups, BDNF levels were higher in the HI+exercise group in comparison to the sham and sham/EX groups (*P* < 0.01). Also, BDNF levels considerably greater in the HI+exercise group in comparison to that in the HI group (*P* < 0.0001) in male rats.

#### 3.1.2. The Impacts of Maternal Exercise through Pregnancy on TNF-*α* Levels of Pups after HI

**Figure 2:**
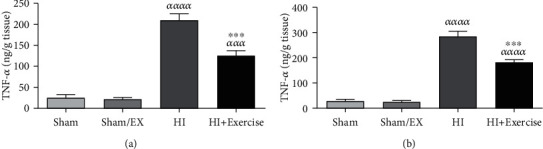
Effect of exercise during pregnancy on TNF-*α* levels after HI in female (a) and male (b) pups. HI: hypoxia-ischemia. *^ααα^P* < 0.001, *^αααα^P* < 0.0001 as compared with the sham and sham/EX. ^∗∗∗^*P* < 0.001 as compared with the HI. Data are expressed as the mean ± S.E.M. (*n* = 10).

According to Figure 2(a), it is indicated that TNF-*α* levels were higher in the HI group in comparison to the sham group and sham/EX groups (*P* < 0.0001). Furthermore, TNF-*α* levels were higher in the HI+exercise group compared to the sham and sham/EX groups (*P* < 0.001) in female pups. Besides, TNF-*α* levels were decreased in the HI+exercise group in comparison to the HI group (*P* < 0.001) in female pups. Based on Figure 2(b), TNF-*α* levels were higher in the HI group in comparison to the sham and sham/EX groups (*P* < 0.0001) in male pups. Furthermore, TNF-*α* levels were higher in the HI+exercise group in comparison to the sham and sham/EX groups (*P* < 0.0001) in male pups. TNF-*α* levels were lower in the HI+exercise group in comparison to the HI group (*P* < 0.001) in male pups.

#### 3.1.3. The Impacts of Maternal Exercise through Pregnancy on CRP Levels of Rat Pups after HI

**Figure 3:**
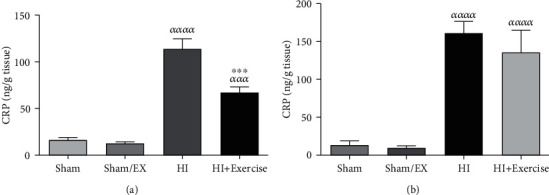
Effect of exercise during pregnancy on C-reactive protein (CRP) levels in pups brain after HI of female (a) and male (b) pups. HI: hypoxia-ischemia. *^ααα^P* < 0.001, *^αααα^P* < 0.0001 as compared with the sham and sham/EX. ^∗∗∗^*P* < 0.001 as compared with the HI. Data are expressed as the mean ± S.E.M. (*n* = 10).

Due to Figure 3(a), CRP levels were higher in the HI group compared to the sham and sham/EX groups (*P* < 0.0001) in female pups. Furthermore, CRP levels were greater in the HI+exercise group compared to the sham and sham/EX groups (*P* < 0.001) in female pups. Besides, CRP levels were reduced in the HI+exercise group compared to the HI group (*P* < 0.001) in female pups. Due to Figure 3(b) in male pups, CRP levels were higher in the HI group in comparison to the sham and sham/EX groups (*P* < 0.0001). Furthermore, CRP levels were higher in the HI+exercise group compared to the sham and sham/EX groups (*P* < 0.0001) in male pups. Besides, there was a nonsignificant difference between the HI+exercise group in comparison to the HI group (*P* > 0.05).

#### 3.1.4. The Impacts of Maternal Exercise through Pregnancy on TAC Levels of Rat Pups after HI

**Figure 4:**
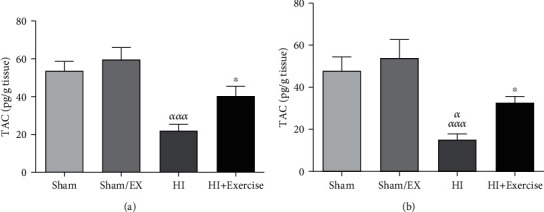
Effect of exercise during pregnancy on TAC levels in female (a) and male (b) pups after HI. HI: hypoxia-ischemia. *^ααα^P* < 0.001 as compared with the sham and sham/EX. ^∗^*P* < 0.05 as compared with the HI. Data are expressed as the mean ± S.E.M. (*n* = 10).

According to Figure 4(a), TAC levels were lower in the HI group in comparison to the sham and sham/EX groups (*P* < 0.001) due to Figure 4(a) in female pups. Furthermore, there was a nonsignificant difference between the HI+exercise group in comparison to the sham and sham/EX groups (*P* > 0.05) in female pups. TAC levels were higher in the HI+exercise group in comparison to the HI group (*P* < 0.05) in female pups. Considering Figure 4(b) in male pups, TAC levels were lower in the HI group in comparison to the sham and sham/EX groups (*P* < 0.001). Furthermore, there was a nonsignificant difference between the HI+exercise group in comparison to the sham and sham/EX groups (*P* > 0.05) in male pups. Besides, TAC levels were decreased in the HI+exercise group in comparison to the HI group (*P* < 0.05) in male pups.

#### 3.1.5. The Impacts of Maternal Exercise through Pregnancy on TOS Levels of Rat Pups after HI

**Figure 5:**
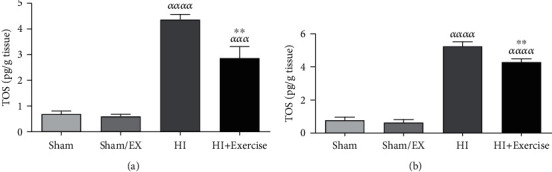
Effect of maternal exercise during pregnancy, on TOS levels in female (a) and male (b) pups after HI. HI: hypoxia-ischemia. *^ααα^P* < 0.001, *^αααα^P* < 0.0001 as compared with the sham and sham/EX. ^∗∗^*P* < 0.01 as compared with the HI. Data are expressed as the mean ± S.E.M. (*n* = 10).

According to Figure 5(a), TOS levels were higher in the HI group in comparison to the sham and sham/EX groups (*P* < 0.0001) in female pups. Furthermore, TOS levels were greater in the HI+exercise group in comparison to the sham and sham/EX groups (*P* < 0.001) in female pups. Besides, TOS levels were lower in the HI + exercise group in comparison to the HI group (*P* < 0.01) in female pups. Following Figure 5(b) in male pups, TOS levels were higher in the HI group in comparison to the sham and sham/EX groups (*P* < 0.0001). Furthermore, TOS levels were greater in the HI+exercise group in comparison to the sham and sham/EX groups (*P* < 0.0001) in male pups. Besides, TOS levels were lower in the HI+exercise group in comparison to the HI group (*P* < 0.05) in male pups.

### 3.2. The Impacts of Maternal Exercise through Pregnancy on Bax and Bcl-2 Expressions of Rat Pups after HI

**Figure 6:**
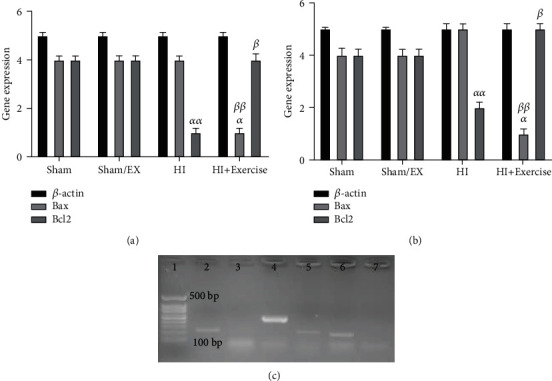
Effect of maternal exercise during pregnancy, on Bax and Bcl-2 gene expressions in female (a) and male (b) pups after HI. (c) Representative reverse transcriptase-polymerase chain reaction of *β*-actin, Bcl-2 and Bax in different groups. (1) Negative control, (2) Bcl-2, (3) Bax, (4) *β*-actin, (5) Bcl-2, (6) Bax, and (7) *β*-actin. HI: hypoxia-ischemia. *^α^P* < 0.01 as compared with HI, and *^αα^P* < 0.05 as compared with the sham and sham/EX. *^β^P* < 0.05 as compared with the HI, and *^ββ^P* < 0.01 as compared with the sham and sham/EX. Data are expressed as the mean ± S.E.M. (*n* = 10).

Due to the results from Figure 6(a), in female pups which their mothers exercised through pregnancy (HI+exercise group), the Bax gene expression revealed considerable differences (decreased) compared to the sham group (*P* < 0.01). Furthermore, in female pups that their mothers exercised through pregnancy, Bax gene expression revealed a considerable difference compared to the HI group (*P* < 0.05). Besides, the gene expression of Bcl-2 in the HI group revealed a considerable difference compared to the sham group (*P* < 0.01) in female pups. Due to Figure 6(b), in male pups which their mothers exercised through pregnancy, Bax gene expression revealed considerable differences (decreased) compared to the sham group (*P* < 0.01). Moreover, he gene expression of Bcl-2 in the HI group showed a considerable differences compared to the sham group (*P* < 0.05) in female pups.

### 3.3. The Impacts of Maternal Exercise through Pregnancy on Infarction Volume and Edema of Brain of Rat Pups after HI

**Figure 7:**
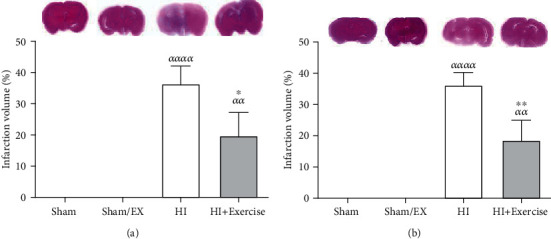
Effect of maternal exercise during pregnancy, on the infarction volume of female (a) and male (b) pups. HI: hypoxia-ischemia. *^αα^P* < 0.01, *^αααα^P* < 0.0001 as compared with the sham and sham/EX. ^∗^*P* < 0.05, ^∗∗^*P* < 0.01 as compared with the HI. Data are expressed as the mean ± S.E.M. (*n* = 10).

Due to Figure 7(a), in female pups, infarction volume was lower in the HI+exercise group in comparison to the HI group (*P* < 0.05) in female pups. Due to Figure 7(b), in male pups, infarction volume was lesser in the HI+exercise group in comparison to the HI group (*P* < 0.01) in male rats.

**Figure 8:**
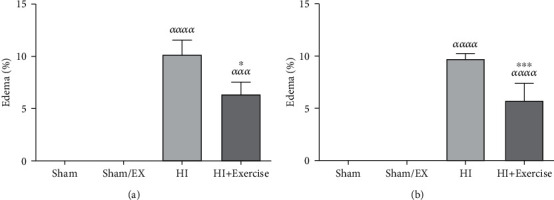
Effect of maternal exercise during pregnancy, on edema in the HI brain of female (a) and male (b) rat newborns. (c) HI: hypoxia-ischemia. *^ααα^P* < 0.001, *^αααα^P* < 0.0001 as compared with the sham and sham/EX. ^∗^*P* < 0.05, ^∗∗∗^*P* < 0.001 as compared with the HI. Data are expressed as the mean ± S.E.M. (*n* = 10).

Due to Figure 8(a), in female pups, edema was lesser in the HI+exercise group in comparison to the HI group (*P* < 0.05) in female rats. Considering Figure 8(b), in male pups, edema was lower in the HI+exercise group in comparison to the HI group (*P* < 0.001) in male rats.

### 3.4. The Impacts of Maternal Exercise through Pregnancy on Cliff Avoidance Test and Negative Geotaxis Test of Rat Pups after HI

#### 3.4.1. Negative Geotaxis Test

**Figure 9:**
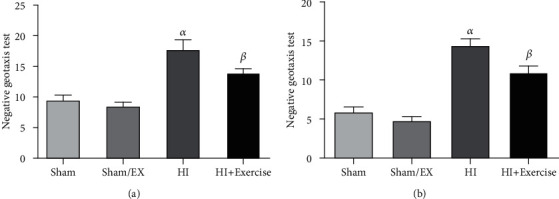
Negative geotaxis test of male (a) and female (b) in sham, HI, and HI/exercise groups. (a) *^α^P* < 0.001, HI/exercise versus sham and sham/EX; *^ß^P* < 0.0001, HI/exercise versus HI. (b) *^α^P* < 0.05 HI versus sham and sham/EX; *^ß^P* < 0.01, HI/exercise versus HI (mean ± S.E.M., *n* = 20).

As indicated in Figure 9, latency in the HI and HI+exercise groups was longer than the sham and sham/EX (both *P* < 0.0001) groups in female pups. Latency in the HI+exercise group was significantly shorter than the HI group (*P* < 0.001).

In male pups, latency in the HI and HI+exercise groups was longer than the sham and sham/EX (both *P* < 0.01) groups in female pups. Latency in the HI+exercise group was significantly shorter than the HI group (*P* < 0.05).

#### 3.4.2. Cliff Avoidance Test

**Figure 10:**
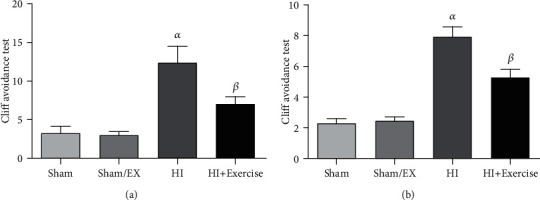
Cliff avoidance test of male (a) and female (b) in sham, HI, and HI/exercise groups. (a) *^α^P* < 0.05, HI versus sham and sham/EX; *^ß^P* < 0.05, HI/exercise versus HI. (b) *^α^P* < 0.001 HI versus sham and sham/EX; *^ß^P* < 0.05, HI/exercise versus HI (mean ± S.E.M., *n* = 20).

As shown in Figure 10, in female pups, latency of the HI and HI+exercise groups was significantly longer than the sham and sham/EX groups (all *P* < 0.05). Latency of the HI+exercise group was significantly shorter as compared to the HI group (*P* < 0.05). In male pups, latency of the HI and HI+exercise groups was significantly longer than the sham and sham/EX groups (all *P* < 0.001). Latency of the HI+exercise group was significantly shorter as compared to the HI group (*P* < 0.05).

## 4. Discussion

Our study highlights the role of exercise during pregnancy in reducing potential injuries due to neonatal HI. The findings of the present study show that treadmill exercise during pregnancy reduces brain damage after neonatal HI by reducing oxidative stress and inflammation as well as increasing the factors preventing cell death.

Other studies have shown the role of exercise in reducing the secretion of inflammatory factors. Wang et al. and Tinius et al. revealed that exercise during pregnancy reduces the release of CRP and maternal systemic inflammation in late pregnancy [14, 15]. Aparicio et al. showed that exercise during pregnancy reduces the release of proinflammatory cytokines such as TNF-*α*, IL-8, and IL-6 in colostrum and mature human milk [16]. Zhu et al. demonstrated that maternal exercise reduced the expression inflammatory factors in the male offspring mice [17].

Inflammation plays an important role in the development of injury after neonatal HI. The results of our study showed that exercise during pregnancy reduced inflammation after neonatal HI.

The pathophysiological mechanisms of many diseases and complications of pregnancy are not well understood. However, studies have shown that oxidative stress is involved in most maternal and fetal complications (e.g., miscarriage, preeclampsia, fetal growth restriction, and preterm labour) [18].

In the present study, treadmill exercise during pregnancy increased TAC. Treadmill exercise in dams through increasing in strength of antioxidant activity of animals weakened oxidative stress after neonatal HI. In concurrent with our results, several human and animal studies have shown that exercise during pregnancy strengthens the antioxidant defense system.

Ramírez-Vélez showed that aerobic exercise training during pregnancy increases antioxidant status in maternal and fetal bloods of nulliparous women [19]. In another study, Ramírez-Vélez et al. demonstrated regular exercise during the second half of pregnancy declines reactive oxygen species production in human placenta [20]. Volpato et al. showed that the swimming exercise model during pregnancy increases SOD and thiol group antioxidants in Wistar rats [21].

Therefore, according to the results of our study and other studies, exercise during pregnancy can reduce the brain damage caused by HI through increasing the antioxidant capacity in pups.

BDNF, as well as its receptor, is expressed in the fetus and plays a vital role in regulating the growth and development of the nervous system [22, 23]. In addition, BDNF is produced by the mother's body and plays an important role in advancing the normal pregnancy process [22]. It can also be transmitted to the fetus through the placenta and is involved in the development of the fetus [22]. Ferrari et al. revealed that exercise during pregnancy led to an increase of 50% BDNF levels in pregnant women compared with controls [24]. Several animal studies showed maternal exercise during pregnancy increases BDNF level in the brain and enhances learning ability in the rat offsprings [25–27]. BDNF enhances the survival of neurons [9, 23]. Studies have shown that BDNF is a neuroprotective agent that reduces neuronal death and the severity of brain damage after HI injury [9]. Also, a higher BDNF level will speed up the healing process after a brain injury [9, 28]. BDNF strengthens the brain's antioxidant system, reduces cell death and infarct size, and enhances sensorimotor recovery after HI injury [9, 28].

The results of our study showed that the offspring of animals who exercised during pregnancy had higher levels of BDNF and antioxidant capacity and less brain damage after HI as compared controls. Studies have shown that exercise increases the expression of antiapoptotic genes, especially increasing the expression of antiapoptotic Bcl and at the same time decreasing Bax genes, which in the nervous system reduces apoptosis and increases the survival of nerve cells [29, 30]. In this way, exercise training can reduce the death of brain cells after HI injury [29].

BDNF increases the expression of the antiapoptotic Bcl-2 protein [31, 32], and thereby exerting part of its neuroprotective effects against brain injuries [32].

In our study, exercise during pregnancy decreased Bax expression and increased Bcl-2 and BDNF levels. Therefore, exercise during pregnancy may reduce apoptosis and cell death after HI injury through increasing the activity of BDNF-Bcl-2 pathways in the pups' brain.

As a result, it seems rational that pups whose mothers exercised during pregnancy had lower infarct volume and better neurological scores after HI injury.

## 5. Conclusion

In the present study, treadmill exercise during pregnancy reduced susceptibility to HI injury in pups. This effect may be mediated by increasing the level of BDNF, Bcl-2 expression, and antioxidant capacity and reducing level of inflammation and oxidant generation which led to lower infarct volume and neurological injury. Finally, it is possible that neonates whose mothers exercised during pregnancy, if exposed to HI condition, have lesser brain injury compared to neonates with no exercised mothers.

However, the present study had some limitations, including pups' recovery and neurological functions not being followed up in the adult period. These limitations attenuate our ability to generalize conclusions about the effects of treadmill exercise during pregnancy on recovery after neonatal HI.

Therefore, it is suggested that in future studies examining the effect of treadmill exercise during pregnancy on the recovery after neonatal HI injury in adulthood is needed.

## Figures and Tables

**Table 1:** Primer pairs and annealing temperatures for reverse transcriptase-polymerase chain reaction.

Gene	Reference	Primer sequence (5′-3′)	Annealing (°C)
Bcl-2	13	F: CTGGTGGACAACATCGCTCTG R: GGTCTGCTGACCTCACTTGTG	63
Bax	13	F: TCCACGATCGAGCAGA R: AAGTAGAAGAGGGCAACC	52
*β*-Actin	13	F: ATTGTAACCAACTGGGACG R: TTGCCGATAGTGATGACCT	55

## Data Availability

The data used to support the findings of this study are available from the corresponding author upon request.
